# Supplementary material for: Integrated analysis of the aqueous humor microbiome and lens capsule transcriptome in high myopia cataract: a pilot study
Source: Front Med (Lausanne). 2026 Jun 16;13:1845205. doi: 10.3389/fmed.2026.1845205 (PMC13314463; doi:10.3389/fmed.2026.1845205)
Supplement: Supplementary file 3 [file Data_Sheet_1.zip › 4.Meta_Statistic/C372089/LEfSe/LEfSe_table.cladogram.pdf]

Cladogram

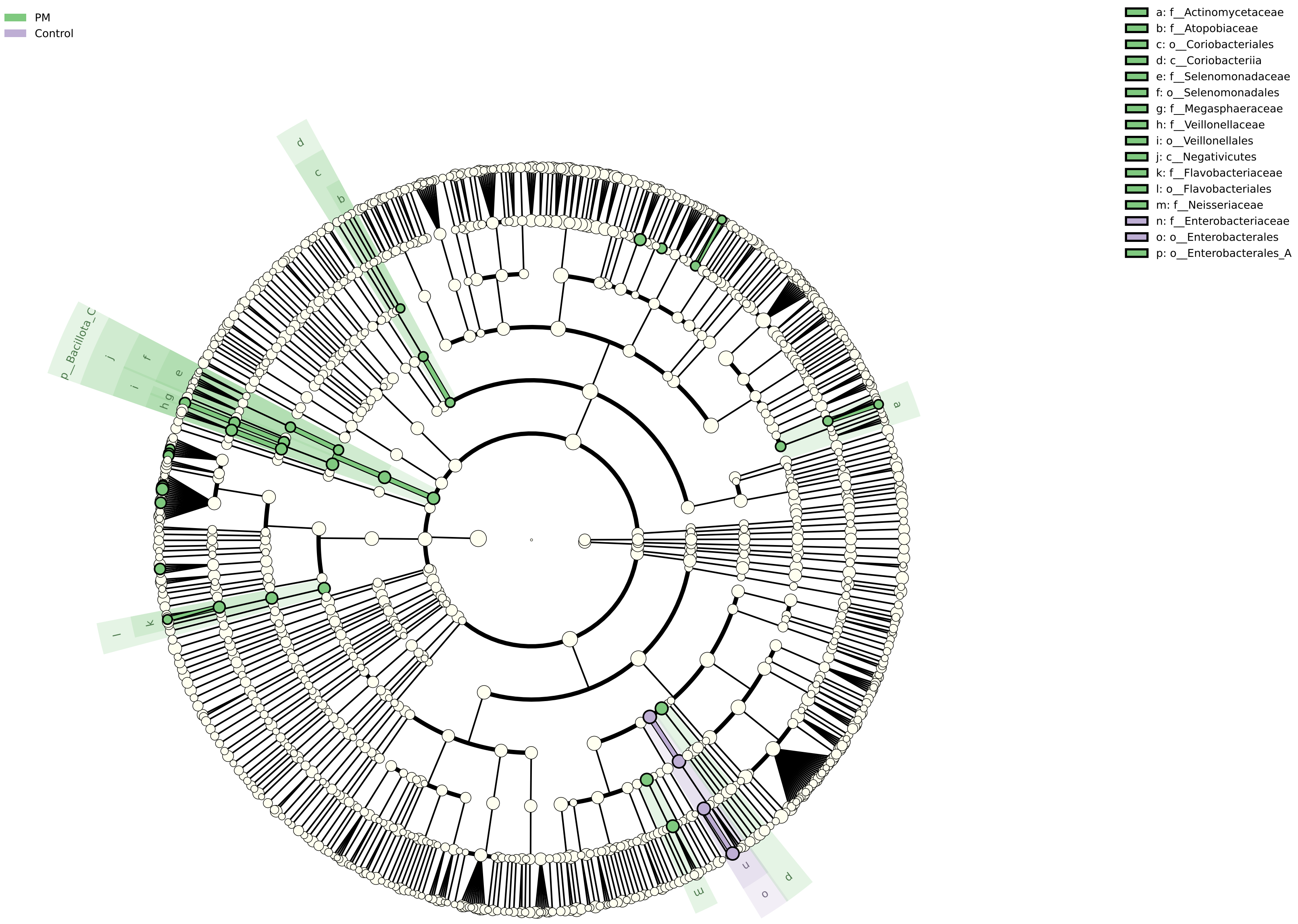
PM
Control
a: f__Actinomycetaceae
b: f__Atopobiaceae
c: o__Coriobacteriales
d: c__Coriobacteriia
e: f__Selenomonadaceae
f: o__Selenomonadales
g: f__Megasphaeraceae
h: f__Veillonellaceae
i: o__Veillonellales
j: c__Negativicutes
k: f__Flavobacteriaceae
l: o__Flavobacteriales
m: f__Neisseriaceae
n: f__Enterobacteriaceae
o: o__Enterobacterales
p: o__Enterobacterales_A
p__Bacillota_C
